# Emerging communities of child-healthcare practice in the management of long-term conditions such as chronic kidney disease: qualitative study of parents’ accounts

**DOI:** 10.1186/1472-6963-14-292

**Published:** 2014-07-07

**Authors:** Ian Carolan, Trish Smith, Andy Hall, Veronica M Swallow

**Affiliations:** 1 School of Nursing, Midwifery and Social Work, Faculty of Medical and Human Sciences, Manchester Academic Health Sciences Centre, University of Manchester, Oxford Road, Manchester M13 9PT, UK; 2 Royal Manchester Children’s Hospital, Central Manchester University Hospitals NHS Foundation Trust, Oxford Road, Manchester M13 9WL, UK; 3 School of Nursing, Midwifery and Social Work, University of Manchester, Oxford Road, Manchester M13 9PL, UK

**Keywords:** Social Learning theory, Self-efficacy, Developing internet resources, Chronic kidney disease (CKD), Children and young people, Parent, Knowledge, Psychosocial support, Communities of practice

## Abstract

**Background:**

Parents of children and young people with long-term conditions who need to deliver clinical care to their child at home with remote support from hospital-based professionals, often search the internet for care-giving information. However, there is little evidence that the information available online was developed and evaluated with parents or that it acknowledges the communities of practice that exist as parents and healthcare professionals share responsibility for condition management.

**Methods:**

The data reported here are part of a wider study that developed and tested a condition-specific, online parent information and support application with children and young people with chronic-kidney disease, parents and professionals. Semi-structured interviews were conducted with 19 fathers and 24 mothers who had recently tested the novel application. Data were analysed using Framework Analysis and the Communities of Practice concept.

**Results:**

Evolving communities of child-healthcare practice were identified comprising three components and several sub components: (1) *Experiencing* (parents making sense of clinical tasks) through Normalising care, Normalising illness, Acceptance & action, Gaining strength from the affected child and Building relationships to formalise a routine; (2) *Doing* (Parents executing tasks according to their individual skills) illustrated by Developing coping strategies, Importance of parents’ efficacy of care and Fear of the child’s health failing; and (3) *Belonging/Becoming* (Parents defining task and group members’ worth and creating a personal identity within the community) consisting of Information sharing, Negotiation with health professionals and Achieving expertise in care. Parents also recalled factors affecting the development of their respective communities of healthcare practice; these included Service transition, Poor parent social life, Psycho-social affects, Family chronic illness, Difficulty in learning new procedures, Shielding and avoidance, and Language and cultural barriers. Health care professionals will benefit from using the communities of child-healthcare practice model when they support parents of children with chronic kidney disease.

**Conclusions:**

Understanding some of the factors that may influence the development of communities of child-healthcare practice will help professionals to tailor information and support for parents learning to manage their child’s healthcare. Our results are potentially transferrable to professionals managing the care of children and young people with other long-term conditions.

## Background

Childhood chronic kidney disease stage 3–5 (CKD) is a complex set of disorders with multiple causes and complications; patients have the condition for life, and optimal management which is essential, requires skilled, home-based clinical care by parents, supported remotely by hospital-based professionals [1]. In previous research [2] parents identified a need for online information and support to supplement, not replace, the support they already received from the hospital-based multidisciplinary team (MDT). It is clear that increasing internet use by society [3-9] has transformed NHS clients’ relationships with information making it more likely parents will search on-line for supplementary care-giving information, although unreliable, misleading and inaccurate sites exist and users must navigate through myth and hearsay [10]. Changing trends in internet use, a dearth of research on what parents think about existing formats of on-line information and support and a ‘digital divide' between those who do/do not have internet access [6] means, therefore, we need a detailed examination of what on-line support parents use, would like, and how they and their children think it could best be delivered to meet parents' variable needs [7,8].

This paper reports on one aspect of a larger study that defined, developed and tested a CKD-specific Internet Health Communication Application (IHCA), the Online Parent Information and Support (OPIS) application. Qualitative interviews with parents were conducted after they had ‘road tested’ OPIS for 20 weeks. The results describe and discuss data emerging from these interviews. Data were analysed using Wenger’s social learning theory, Communities of Practice (CoP) [11-17]. Learning occurs between group members engaged in joint enterprise to formulate a common set of solutions to familiar problems [13]. Communities of practice members share the same goals, interests, and repertoire of resources [11]; they draw on shared methods of "doing and undertaking tasks" to achieve their goals, using the same tools and common language to produce a unique body of knowledge [15,18]. The CoP becomes the educator and/or educational environment [16].

Lesser & Storck [17] suggest CoPs generate social capital between members, creating organisational resources and interaction guidance. Community interactions create enhanced knowledge, trust, reciprocity, co-operation and behavioural changes that strengthen social capital and enhance CoP performance [19-21]. These relationships do require nurturing to ensure a CoP’s effective function [22]. Members of CoPs ‘problem solve’ by using the principles of social capital: shared language, shared experiences, self-development, mutual trust, and identification with the community [23] to enhance the learning process. Flora [24] contends that CoPs best solve problems when they are diverse, inclusive, flexible, horizontal (linking with those of similar status), and vertical (linking with those of different status).

The flexibility of the CoP concept makes it ideal to adapt to healthcare management; these communities generate ideas for new services, practices and products [25]. Communities of Practice address complex dilemmas such as improving quality and safeguarding high standards of care by fostering an environment for clinical care excellence [26]. Therefore, the CoP concept has learning potential for health professionals [14,27-30] and parents. After conducting an extensive library search no published material citing Communities of Practice using parent perspectives in child-health care to elucidate the theory was found.

Le May [14] suggests that CoPs generate professional and human/patient capital. Participants transfer socio-cultural practices between group members, who then contribute to the overall development of capital for that community. Patients and families living with childhood long-term conditions such as CKD, develop capital by: understanding the way people view them or their child’s health; expressing satisfaction and expectation and contributing to changing healthcare services. Generating capital creates a store used by others, including patients, parents and professionals. Parent and patient story telling is increasingly important in enhancing health professional’s knowledge capital as they learn from stories [2,14,31]. The evolution of patient/family and care centred CoPs and how they develop capital is, therefore, fertile research ground [2,32]. For the purposes of this paper we used a conceptual framework that to our knowledge has not previously been used in this context. It illuminates parents’ views and experiences of using OPIS in relation to their involvement with health professionals as they shared management of their child’s CKD. The published protocol for the wider study can be found at [33].

## Methods

The study was approved by NHS Research Ethics Committee 11/NW/0268.

### Research setting

The study was based in a large Children’s Hospital in the North West of England; one of the thirteen regional centres caring for UK children with CKD, and specialising in dialysis and transplantation. Families from different UK regions access the centre.

### Sampling and recruitment

The target population were parents of children aged 0–18 years with CKD whose care was being managed in this centre, using a purposive sampling approach based on parents’ ethnicity, age, gender and their child’s CKD stage. Forty three parents provided written consent and participated in semi-structured interviews (Table 1).

**Table 1:** Sample characteristics

**Category**	**CKD 3**		**CKD 4-5**		**Total**
	**White European/Afro Caribbean**	**South Asian**	**White European/Afro Caribbean**	**South Asian**	
**Gender**					
Female	7	0	15	2	24
Male	3	0	10	6	19
**Family details**					
Single parent interviews	4	0	13	4	21
Couple parents interviews	3	0	6	2	11
**Age**					
16-24	0	0	1	0	1
25-49	6	0	16	8	30
50-64	4	0	8	0	12
65+	0	0	0	0	0

### Data collection and analysis

Individual, semi structured qualitative interviews were undertaken by IC using a topic guide [34]. Interviews took place in parents’ homes and occasionally were supplemented by telephone interviews [35], or a quiet room in the hospital according to participants’ preferences. The interviews lasted 45 minutes on average and were digitally recorded, transcribed and later analysed by IC and VS using Framework, thus providing auditable, organised data management and transparency of findings. Transcriptions were read and emerging themes were entered into a chart and then attributed a hierarchy based upon their connectivity. After further reading, emerging themes and sub themes iteratively altered the charting process, the data were finally added to the Framework before review by the study team [36-39].

## Results

### Sample characteristics

A total of 43 parents (19 fathers and 24 mothers of 32 children) were interviewed in11 couple and 21 individual interviews.

### Findings

Parents’ accounts in our previous research suggest ‘common ground’ [18] exists between themselves and health professionals; in the present study it emerged that this common ground can also contribute to the development of communities of child-healthcare practice (CoC-HP); these evolve iteratively not linearly due to frequent dynamic health care challenges. Parents act as CoC-HP co-ordinators [12] developing community capital by exchanging knowledge between members. Parents’ data demonstrate components and sub-components that correspond with the CoP components defined by Wenger: *Experiencing, Doing, and Becoming/Belonging*[11,12] and also illuminates specific factors that affect CoC-HPs’ development. These findings are presented below in composite case studies. The ellipsis in brackets (…) signifies omitted text; square brackets denote explanatory text. The components and sub-components of the CoC-HP are:

Experiencing (see Additional file 1 for relevant composite case study)

Parents often experience shock and trauma when learning of their child’s CKD; they seek to negotiate its meaning so they can develop strategies that aid the incorporation of health care into daily routines.

#### Normalisation of care

Parents accommodate health care procedures into their daily lives, altering their family routine in order to learn the procedures, thereby ensuring that family life runs as smoothly as possible. Parents spend significant chunks of time delivering clinical care and it can make them feel they are neglecting other siblings. Embedding care into family routine helps to ensure that siblings know that care is necessary yet the patient is not favoured by their parent[s].

#### Normalisation of illness

Parents try to prevent their ill child being defined by their condition, so they do not become a caricature of illness. Most parents instil mental resilience in their child enabling them to integrate the illness into their lives. They encourage their child to develop coping strategies to ensure the effects of CKD do not negatively impact on their child’s experience of life opportunities, and enabling them to do the things that ‘well’ children do.

#### Acceptance and action

Parents reported that acceptance is crucial for themselves to integrate CKD into their lives.

#### Gaining strength from the affected child

Parental mental resilience to CKD is affected by their child’s reaction to receiving health care. Clinical procedures can be invasive, time consuming and stressful for parents and children; parents reported being distressed by their child’s discomfort and pain, however if the child copes, parent(s) are comforted by this and gain strength from it.

#### Building relationships to formalise a routine

The many types of relationships parents discussed included learning to work with: the MDT at the hospital, General Practitioners and community health care staff; and developing empathetic relationships with neighbours, friends and members of the extended family who have personal health care experience. These relationships help parents to develop a CoC-HP that assists in managing their child’s care.

Additional file 1 presents a composite case study drawn from our data illustrating sub components and related quotations reflecting the way parents experience the components of the CoC-HP.

Doing (see Additional file 2 for relevant case study quotations).

This component concerns the active part of parents’ caring for their child’s clinical needs; learning through joint enterprise and shouldering the responsibilities and burden of healthcare. Three subcomponents help to illustrate this (see example quotations, Additional file 2).

#### Developing coping strategies

Parents said they learn to cope with the responsibility of care by seeking activities to positively channel negative emotions; including, going to the gym, going for a run, discussing issues with friends at social meetings, gardening and comparing their child’s condition with children in worse health. They were comforted by not being alone or the realisation that they are not the most unfortunate family using the centre.

#### The importance of Parent’s efficacy of care

Many parents take the responsibility of clinical care very seriously and want to demonstrate health care competence within their CoC-HP to ensure their child receives the best possible care and achieves a managed ‘wellness’. Parents find it difficult to delegate clinical procedures to others in their family support network due to the burden of managing wellness and because they know their own child better than anyone else.

#### Fear of the Child’s failing health

Children’s health status can fluctuate widely which can be exceptionally stressful for them yet also be a motivator for parental care efficacy. Parents want to minimise episodes of poor health for their child as much as possible.

Additional file 2 uses a composite case study drawn from the data to illustrate sub components and related quotations reflecting the way parents actively care for their child’s CKD, thereby demonstrating the ‘Doing’ component of the CoC-HP.

Belonging/Becoming (see Additional file 3 for relevant quotations).

As CoC-HP co-ordinators, parents have built considerable contextual and care knowledge capital regarding their child; this capital spreads throughout the CoC-HP, cementing parents’ identity as CoC-HP co-ordinators; they now exude an accomplished and confident demeanour. However, due to the changing nature of CKD, the CoC-HP, it’s capital and parental identities are likely to be fractured and rebuilt according to the emerging care needs of the child.

#### Information sharing

Experienced parents feel confident sharing their contextual care knowledge with other parents; filtering this knowledge helps them to fill gaps between MDT endorsed clinical approaches and parents’ and/or families’ care contexts. Advice from other experienced parents provides tacit knowledge for families who are negotiating meaning in their child’s care. Information sharing creates capital between families and widens individual CoC-HP membership.

#### Negotiating with NHS staff

In the ‘experiencing’ component parent data suggests that they find it difficult to challenge staff in their CoC-HP if they have concerns about clinical issues, because they rely on MDT members’ experience and knowledge capital. In time, however, parents develop their own experience and knowledge within their CoC-HP, thereby benefiting from their own contextual insight of their child. This insight helps parents to feel confident enough to negotiate with staff about their child’s health care issues such as: administering medication; performing clinical procedures; instilling a clinical regimen and disciplining their child.

#### Expertise in care

Regarding the administration of complex care procedures parents view their competency as progressing to expertise. For example, when replacing urinary catheters or naso-gastric tubes, and ensuring their child adheres to their medication regimen, they know that by becoming experts they are helping to relieve their child’s pain and suffering.

Additional file 3 uses a composite case study to illustrate sub components and related quotes reflecting the way parents’ feel as if they are beginning to belong to the CoC-HP and construct a care identity.

Although the CoC-HP components we identified in our data (Experiencing, Doing and Belonging/Becoming) demonstrate the evolution of this community; the dynamic nature of CKD means progression is achieved iteratively not linearly. Parents accounts also indicate seven key factors that affect a CoC-HP’s development.

Factors Affecting the Development of the CoC-HP.

#### Service transition

Parents’ suggested that they fear change in the child’s clinical circumstances; fear of the unknown adds stress, when parents have become accustomed to specific routines this helps them to normalise CKD management into family life, change is therefore, a concern for them. Transitions, such as transferring from children’s to adult services, or from treatment with dialysis to kidney transplantation means that new community members and knowledge capital must be integrated and new meaning negotiated within the CoC-HP to ensure it functions effectively.

#### Poor parent social life

The intense health care needs of children with CKD are time consuming; these demands affect parents’ earning potential as they fit employment around their child’s healthcare. Leisure time is eroded when CKD care and paid employment are crucial. However, leisure time helps parents to process the burden of managing care. Care responsibility often resides with parent(s) alone, due to its responsibility, family members or friends who might usually help share child care with the parents, therefore lack the necessary clinical skills, thus reinforcing the parental burden of responsibility.

#### Psycho-social effects on parents

Parents are affected by: the trauma of finding out that their child has CKD; the variable nature of care management; communication and relationship breakdown; disparity in health care management between parents; and the fear for siblings’ welfare due to the significant time spent with the patient. Some parents who are experienced at successfully managing their child’s health care felt it strengthened their family, but recognised that health care should not be the sole aim of family life. Parents recommended that the opportunity to discuss these issues with a social worker and/or clinical psychologist would help them to process these psycho-social issues.

#### Family chronic illness

Some families have more than one member experiencing chronic illness; this affects how families cope with care management and how their CoC-HP evolves.

### Difficulty in learning New procedures

CKD management procedures are complex, requiring consistent and detailed MDT support. The critical nature of delivering clinical procedures means parents have to get care right each time. There can be strong resistance and reservations from parents about their ability to administer care procedures.

#### Shielding/avoidance

In some cases parents limit sharing knowledge with their partner to shield them from distress caused by their child’s deteriorating health. In addition, some parents avoid facing the reality of their child’s condition, blaming other factors; this causes dysfunction in the CoC-HP affecting care efficacy and the managed wellness of the child.

#### Language and cultural barriers

In families where English is a second language, clinical messages may be misunderstood by parents and consequently a child’s clinical care and long term clinical outcomes could be negatively affected. Cultural traditions may also bar specific types of care or donation of kidneys to improve patient health and wellbeing thus causing dysfunction in the development of the CoC-HP. MDTs may exacerbate this if they lack knowledge of local languages, therefore limiting the knowledge capital they can share with these parents, especially if interpreters need to be included in the CoC-HP.

Additional file 4 uses a composite case study to help illustrate sub components and related quotations reflecting the way the CoC-HP evolves.

## Discussion

**Figure 1:**
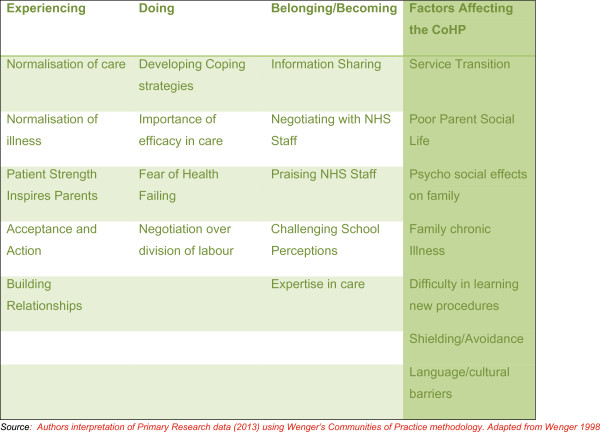
The community of child-healthcare practice model.

The data presented here are significant because families are expected to execute complex, home-based clinical care procedures that require high levels of competence [30,32,40-44]; failure to safely carry out this care could affect their child’s clinical outcomes. However, there is a dearth of collaboratively developed, evidence based information to address their parents’ needs and preferences. There is also a lack of guidance for professionals on the factors that influence the development of communities of child-healthcare practice [2]. Three main components of CoPs as defined by Wenger, when integrated with the factors that affect development of the community have resulted in the Community of Child-healthcare Practice Model (Figure 1).

Connections between the CoP components of Experiencing, Doing and Becoming/belonging and the factors that influence development of communities of healthcare practice as identified by parents provide a conceptual framework that acts as a foundation to inform the development of parent information and support by healthcare professionals as they share responsibility for condition management.

The analysis presented here builds on previous research and helps to elucidate the components of CoC-HPs through parents’ accounts of their child’s care management [40], also revealing factors affecting the function of a CoC-HP.

When discussing ‘*normalisation of illness* and *care’* and ‘*acceptance and action’* parents in our study demonstrated a desire to manage, control, integrate change and create meaning early in their child’s CKD trajectory in order to incorporate the condition into their daily routine [2]. There are similarities between this analysis and the ‘Experiencing’ component of Wenger’s CoP concept; Wenger [11,12] suggests that negotiating meaning (how we experience and engage with the world) requires sustained attention and readjustment by participants to achieve accomplishment. In our study, parents’ data described a CoC-HP sub component of ‘*Gaining strength from their child’* that cements this hypothesis. Although children with CKD often require invasive and painful clinical procedures [1] the child’s reaction to these procedures helped parents to negotiate meaning from it, seeing their child’s health improve sometimes acted as a catalyst providing parents with the mental and physical stamina necessary for complex care management. Lave and Wenger [13] similarly find that learners begin their journey on the periphery of CoPs and move towards full participation as they negotiate their own place within that community. In the same way, when discussing ‘*Building relationships’* parents’ accounts illuminated another feature of Lave and Wenger’s [13] assertion that community members are trying to negotiate meaning by learning from individuals they feel provide situated experience.

In the CoC-HP, parents talked of *‘Developing coping strategies’* to relieve the significant burden of care management [31,41-45]. Wenger [12] asserts that when there is too much dependence on the activity of a co-ordinator, in this case parent(s) act as CoC-HP coordinators, this burden of responsibility makes the CoC-HP vulnerable to dysfunction. Parents in our study, therefore, appeared to be remedying potential CoC-HP dysfunction by recognising the necessity to alleviate their burden of responsibility, learning to cope by implementing strategies. In addition, parents revealed the *‘Importance of care efficacy’ and ‘Fear of failing health’* which is akin to Wenger’s assertion [12] that co-ordinators of an enterprise are determined to be competent in it. Practice leads to competence in the enterprise and parents’ data revealed a determination to avoid CoC-HP dysfunction by achieving care efficacy that would contribute to a ‘managed wellness’ for their child.

Our description of *‘Sharing of Information’* is consistent with a key sub component of Wenger et al’s CoP [13] as they suggest it is essential to avoid community dysfunction. They claim that this is why avoiding complacency and incorporating new members into the CoC-HP are important factors when the community achieves expertise, so that progressive shared learning continues within the CoC-HP. New parents or health professionals providing their knowledge capital help to increase the CoC-HP store of knowledge capital, thus avoiding group stagnation and dysfunction. Health professionals also learn from parents, particularly as they establish their identities as expert carers; the *‘Negotiating with NHS Staff’* sub-component is illuminated by Wenger et al’s [13] sub component suggesting that peace and harmony are not essential in CoPs. It is beneficial if members achieve expertise by challenging and negotiating with each other, it indicates that parents are gaining expertise [2]. It therefore follows that if parents exhibit ‘*Expertise in care*’ as CoC-HP co-ordinators they exude confidence and knowledge and assert their expertise in the CoC-HP because they have learnt from professionals [46] and are confident expressing their identity.

Several factors were also identified in our data that affect CoC-HP development, sometimes causing dysfunction; these are discussed next. The ‘*Service Transition’*; and *‘Learning New Procedures’* sub components occur anywhere within the CKD trajectory [30]. Receiving a different service or learning new tasks, requires the negotiation of meaning and therefore, the *Experiencing* component of the CoC-HP begins again as parents and professionals adapt to the revised CoC-HP context [11], how this is accommodated affects the function of the CoC-HP [12]. The *‘Poor Parent Social Life’* sub-component occurs due to the time consuming care needs of the patient [2], this can lead to parents feeling overloaded. Wenger et al. suggest [12] if co-ordinators of enterprises, in this case parents, are not supported or given opportunities for self-expression it will lead to CoC-HP dysfunction. Parents in turn may display *‘Psycho Social Effects’*; these emerge for many reasons including *‘Family chronic illness’* placing added care burden and responsibility on parents and the extended family, intensifying stress and the potential for CoC-HP dysfunction. It is essential for health professionals to appreciate individual family contexts when they enable the functioning of CoC-HPs [2,18], for example, professionals may identify issues such as chronic sorrow [2,47] affecting parental care management efficacy and leading to CoC-HP dysfunction [12].

Parents occasionally decide to practice ‘*Shielding/Avoidance’* to protect their partner, themselves or family members from upsetting information; they may feel it helps to improve their CoC-HP function by preventing short term panic or hysteria [48]. However, ultimately it leads to complacency due to the clique of support that develops. Wenger et al. [12] identify cliques developing when participants are deliberately marginalised or excluded in the CoP, it leads inevitably to dysfunction. Some parent data revealed a *‘Language/Cultural’* barriers sub component that may exist in areas of cultural diversity; the cultural competence [46] of CoC-HPs’ health care provision may be affected. Health care organisations must provide care to patients with diverse values, beliefs and behaviours, tailoring delivery to meet patients’ social, cultural, and linguistic needs. Barriers to cultural competence include a lack of diversity in health care leadership and workforce; systems of care are poorly designed to meet the needs of diverse patient populations such that poor communication between providers and patients of different racial, ethnic, or cultural backgrounds can exist.

The strengths of this study include; a strong and multi-disciplinary study team comprising of clinical and research professionals and parents that enabled detailed, focused data capture. Face to face interviews helped to elicit greater depth of detail from parents [37]. Our purposive sampling approach meant our results specifically apply to the research cohort because parents from different ethnic and socio economic backgrounds participated, and 19 fathers (a group who are often under-represented in healthcare research) took part in the study [31,49-53]. Parent recollections were first hand experiences of care management; couple interviews enhanced discourse due to this double hermeneutic [54], as participants shared their mutual care experiences. Our analysis of the data gathered is potentially transferrable to CoC-HPs responsible for managing other long term conditions. After a search for the term ‘Communities of Child-healthcare Practice’ using a variety of library search tools, we concluded there is not a parent centred study that observed the abstract components of the COP model elucidating and locating them within empirically observed Communities of Child-healthcare Practice; therefore this paper complements and builds on existing Communities of Clinical [27,28] and Nursing [29,30] Practice literature.

Study limitations include the fact that sampling involved only one UK kidney unit, a sample that accounts for greater diversity of age, gender, cultural differences and socio-economic status would have enhanced the variety and depth of data collected. Data collection relied on verbal recollection of care provision by parents only. Our list of factors affecting CoC-HP development is not exhaustive and it is recommended that further research be carried out to reveal additional factors that exist and their effect on CoC-HP function. Future research could include a longitudinal, ethnographic study whereby researchers observe a range of CoC-HP members (for example, patients, parents, health professionals and family friends) over a period of time, involving a range of chronic childhood conditions in different health care settings.

When considering the CoC-HP components and sub components described and discussed in this paper, it seems prudent to suggest that further research should look at how the CoC-HP model and factors affecting it could be used to enhance health care practice, aiding professionals to further understand parents’ evolving care management needs [2]. Implications of these findings are important as improving services is important [55] and authors [32,56] suggest that future health care funding needs to be directed towards effective, and away from ineffective interventions, and that contextual knowledge is needed. A CoC-HP model such as the one presented here (Figure 1) could, therefore, potentially help health professionals and inform their decisions when supporting parents.

## Conclusions

When discussing their views on OPIS following a trial period, parents also revealed the existence and evolution of CoC-HP’s that focused on their child’s clinical care. Building on the established concept of CoP, our analysis has uncovered several components exhibiting both the evolution of the CoC-HP and also the factors that affect its development. Data revealed that there are distinctive CoC-HPs that evolve along the trajectory of a child’s CKD. This paper discusses the comparisons between CoPs and CoC-HPs and concludes that it is important to learn lessons from CoC-HPs. Important future research questions would address the following: can CoC-HPs be engineered to facilitate effective care?; what additional factors affect the development of CoC-HPs?, how do CoC-HPs evolve in different environments?

### Disclaimer

This paper presents independent research funded by the National Institute for Health Research (NIHR) under its Research for Patient Benefit (RfPB) Programme (Grant Reference Number: PB-PG-0110-21305). The views expressed are those of the authors and not necessarily those of the NHS, the NIHR or the Department of Health. We confirm that all parent identifiers have been removed or disguised so the parents described are not identifiable and cannot be identified through the details of the story.

## Competing interests

We confirm that none of the authors have competing interests.

## Authors’ contributions

IC made a substantial contribution to acquisition of data, coordination of the study, data collection, data analysis and interpretation, leading the drafting of the manuscript, and finalising the version of the manuscript to be published; TS made substantial contribution to setting up the study site, data analysis and interpretation, drafting of the manuscript, and has given final approval of the version to be published; AH participated in design and coordination of the study, made substantial contribution to data interpretation, drafting of the manuscript, and has given final approval of the version to be published; VS conceived of the study, led its design and coordination, made substantial contribution to analysis and interpretation of data, drafting the manuscript, revising it critically for important intellectual content and has given final approval of the version to be published. All authors read and approved the final manuscript.

## Pre-publication history

The pre-publication history for this paper can be accessed here:

http://www.biomedcentral.com/1472-6963/14/292/prepub

## Supplementary Material

Additional file 1 The Glovers, a composite case study. Click here for file

Additional file 2 The Rayhans, a composite case study. Click here for file

Additional file 3 The Bests, a composite case study. Click here for file

Additional file 4 Composite case study demonstrating factors affecting development of the CoHP. Click here for file
