# Supplementary material for: Emerging communities of child-healthcare practice in the management of long-term conditions such as chronic kidney disease: qualitative study of parents’ accounts
Source: BMC Health Serv Res. 2014 Jul 7;14:292. doi: 10.1186/1472-6963-14-292 (PMC4107554; doi:10.1186/1472-6963-14-292)
Supplement: Additional file 1 — The Glovers, a composite case study. [file 1472-6963-14-292-S1.docx]

Additional file 1: The Glovers, a composite case study:

Robert and Rihanna Glover have a 4 year old daughter Kate who was recently diagnosed with CKD and at the time of this research they are making sense of what managing the disease would mean for themselves and their daughter. Robert and Rihanna both work full time and they are trying to incorporate Kate’s disease into their routine. They visit the outpatient clinic to discuss ‘*normalising care’* into their daily routine. Robert says to a nurse, *“… we don't want to make a big issue with this [clinical care], we want to do it at the proper time before she goes to bed and in the mornings if we can”.* Rihanna also mentions that she doesn’t want her child to define herself by her illness and therefore seeks to ‘*normalise the illness’* into their routine also. She says: *everyone wants to spoil her [because of her CKD] and I want her to just be the same as everyone else, because that’ll be another problem then, that’s when she will be naughty”.*

Robert seems to be negotiating this meaning and, therefore, the implications of managing Kate’s CKD, he tells the nurse how they *‘gained strength’* from Kate, they are proud because when her health deteriorated recently she had to go for a biopsy and convalesced on the ward, "*We walk in, she lay there in her bed and she’s got tubes coming everywhere and she’s got her sun glasses on!".* This display of resilience by Kate to what was happening to her helped Rihanna to move towards ‘*acceptance and action’* with the disease. She mentioned *"[Eventually], it [the kidney transplant] will last for years to come and when she has that she doesn't have dialysis, it’ll open things up [improve her quality of life]"*.

Both parents talked about how they’ve been making sense of how to manage the condition by *‘building relationships to formalise a routine’*. They have friends whose father had kidney disease and they seek their advice for tacit knowledge that helps them to negotiate the meaning of their own experience. Rihanna says *“I used to have a close friend and he had the same [disease], his family …live around the corner and his wife comes and talks to me [about her experience] ".*
