# Supplementary material for: Emerging communities of child-healthcare practice in the management of long-term conditions such as chronic kidney disease: qualitative study of parents’ accounts
Source: BMC Health Serv Res. 2014 Jul 7;14:292. doi: 10.1186/1472-6963-14-292 (PMC4107554; doi:10.1186/1472-6963-14-292)
Supplement: Additional file 2 — The Rayhans, a composite case study. [file 1472-6963-14-292-S2.docx]

Additional file 2: The Rayhans, a composite case study

Zara and Iqbal have a son Rohan, aged three, who has been receiving haemodialysis for a year; having been diagnosed with CKD 12 months ago; his parents have been negotiating his care management with each other. Rohan’s kidney function has recently deteriorated and he has been admitted to the renal ward. It’s been a ‘roller coaster ride’ for both parents, as they explain to the play worker who is visiting Rohan. They tell the play worker how they have been ‘*Developing coping strategies’* to ensure that they have personal time to process what they have been thinking about and learning, putting their lives into perspective. Zara says, *“The good leveller is coming here [hospital] every day, if you're having a low moment .go for a walk round, you see children suffering far worse than my son".* It is so important to both parents that they provide ‘*efficacy of care’* to their child. Iqbal reflects on his care management as if his need to control the administration of the medications is somehow under scrutiny. He says, *“..because it is potassium and I know how lethal it is I'd prefer to give it to him myself”.* They have a real *‘fear of his [Rayhan’s] health failing’* but acknowledge that this is all part of Rayhan’s life. Zara says, *“At different stages of the condition… (my) child’s condition improved and deteriorated, [it’s the] ups and downs of the illness”.* Both parents appreciate it is very difficult to get a state of managed wellness for their child but are determined to achieve this by working together with the health professionals and learning from their professionals’ experience.
