# Supplementary material for: Emerging communities of child-healthcare practice in the management of long-term conditions such as chronic kidney disease: qualitative study of parents’ accounts
Source: BMC Health Serv Res. 2014 Jul 7;14:292. doi: 10.1186/1472-6963-14-292 (PMC4107554; doi:10.1186/1472-6963-14-292)
Supplement: Additional file 3 — The Bests, a composite case study. [file 1472-6963-14-292-S3.docx]

Additional file 3: The Best’s, a composite case study

Ronny and Michelle have a daughter called Ellen, aged 14; Ellen has had multiple chronic health problems, including CKD, but her parents are comfortable managing the care of these conditions, although they recognise that new challenges can arise at any moment. The family are attending their bi-monthly appointment with the Consultant Paediatric Nephrologist who greets them with a familiar smile and joke, the nurse specialist is in the clinic room too and asks them if they would mind ‘*Information Sharing’ with another family* with limited experience of managing CKD. Ronny says that it’s no problem and Michelle says, *“When we’re talking with other parents they’ve asked us and [then] said, oh we hadn’t thought of that".* The nurse specialist thanks them and tells them how beneficial it will be for this family who are struggling to incorporate caring for their child’s CKD into their daily routine.

The family share reflections with the health professionals, particularly regarding the last time Ellen had a serious infection. Michelle laughs recalling that she had no problem, *‘Negotiating with NHS staff’*, she said, “*Yeah, I’m not quiet, when it comes to my daughter, I want to know the ins and outs!".* The consultant and nurse specialist explain that is why they want them to speak to other patients because of their confidence in addressing health professionals, always willing to ask questions and obtain the best outcome for their child’s wellness. Ronny then exhibits his and Michelle’s *‘expertise in care’* by saying to the two health professionals, *“Yeah, I mean it's easier now because obviously I suppose we’ve been through the learning phase and we are both relatively bright and we pick things up quite quickly. We’re just used to it I think".* It’s clear from the conversation that the professionals have a rapport with the Bests due to their expertise, thus removing dependency upon the health professionals to teach, and on the parents to learn.
