# Supplementary material for: Emerging communities of child-healthcare practice in the management of long-term conditions such as chronic kidney disease: qualitative study of parents’ accounts
Source: BMC Health Serv Res. 2014 Jul 7;14:292. doi: 10.1186/1472-6963-14-292 (PMC4107554; doi:10.1186/1472-6963-14-292)
Supplement: Additional file 4 — Composite case study demonstrating factors affecting development of the CoHP. [file 1472-6963-14-292-S4.docx]

Additional file 4: Composite case study demonstrating factors affecting development of the CoHP

Donna has a son of 18 called Bradley, they have been struggling to adapt to *‘service transition’,* the inevitability of him transferring to adult services is leading him to refuse treatment and he can, from time to time, curse at his mother and the clinical staff. Donna speaks to a staff nurse, Bill, whilst her son is receiving care on the renal ward. She tells Bill that, *“The transition to adult renal services will be difficult for [my] son”.* She goes onto tell Bill how she can’t find any time for herself and has a *‘poor social life’* because she has three other children, pets and a sick father to care for on top of caring for Bradley. She complains, *“Dialysis is very time consuming and [it’s] placed a burden on [my] leisure time, preventing me from [having] time off”.*

The burden of care has also led to ‘*Psycho-social effects’* on Donna. She says about the hospital, *“You have a lot of medical support but they don’t really have that emotional and social welfare type support at all. It just falls through everybody’s… responsibility… to provide".* Donna feels that having some support to listen to her and perhaps coach her to think of solutions would be really beneficial. She mentions her recent additional *‘family chronic illness’* because her daughter was diagnosed with leukaemia *“I’m pleased that the burden of care will be reduced [eventually] with [my] son’s [kidney] transplant because my daughter is chronically ill also’.* Donna is struggling to cope with the demands of a family and the complex care needs of two of her children, particularly the *‘difficulty in learning new procedures’* regarding her son’s change in dialysis procedure. *"I can’t stand messing with wires and stuff oh no, and plus I’ve got other kids as well".*

Although Donna is worried about adapting she feels there is no alternative to her providing the care. When Bill suggests that one of her eldest children might be taught to deliver the care she practices *‘shielding and avoidance’. “I can be taught by somebody once, but they [eldest children] …are scared, they know that with the machines… something can go wrong and they don't want it.. [the responsibility of operating dialysis machine]".* Bill explains that Donna will have to make a decision to change something in her life because she is struggling and she needs help, he asks if any relatives of Donna’s father might help, Donna says that isn’t possible due to *‘Language and Cultural barriers’, ‘[My ex partner’s] family can’t help due to not speaking English and living quite a distance away’.* All of these factors are affecting the managed wellness of Bradley and the multi-disciplinary team are keeping a close eye on his management. Donna feels lost and is resigned to the negative, downward spiral of CoCP dysfunction.
